# Cerebral inducible nitric oxide synthase protein expression in microglia, astrocytes and neurons in *Trypanosoma brucei brucei*-infected rats

**DOI:** 10.1371/journal.pone.0215070

**Published:** 2019-04-17

**Authors:** Raymond Cespuglio, Donia Amrouni, Elizabeth F. Raymond, Bernard Bouteille, Alain Buguet

**Affiliations:** 1 Neuroscience Research Centre of Lyon (CRNL), Neurochem, Faculty of Medicine, Claude-Bernard Lyon-1 University, Lyon, France; 2 Sechenov 1st Moscow State Medical University, Laboratory of Psychiatric Neurobiology, Moscow, Russia; 3 Faculty of Medicine, team EA 4171, Claude-Bernard Lyon-1 University, Lyon, France; 4 Department of Parasitology, Dupuytren University Hospital, Limoges, France; 5 Malaria Research Unit, UMR 5246 CNRS, Claude-Bernard Lyon-1 University, Villeurbanne, France; Karolinska Institutet, SWEDEN

## Abstract

To study the anatomo-biochemical substrates of brain inflammatory processes, Wistar male rats were infected with *Trypanosoma brucei brucei*. With this reproducible animal model of human African trypanosomiasis, brain cells (astrocytes, microglial cells, neurons) expressing the inducible nitric oxide synthase (iNOS) enzyme were revealed. Immunohistochemistry was achieved for each control and infected animal through eight coronal brain sections taken along the caudorostral axis of the brain (brainstem, cerebellum, diencephalon and telencephalon). Specific markers of astrocytes (anti-glial fibrillary acidic protein), microglial cells (anti-integrin alpha M) or neurons (anti-Neuronal Nuclei) were employed. The iNOS staining was present in neurons, astrocytes and microglial cells, but not in oligodendrocytes. Stained astrocytes and microglial cells resided mainly near the third cavity in the rostral part of brainstem (periaqueductal gray), diencephalon (thalamus and hypothalamus) and basal telencephalon. Stained neurons were scarce in basal telencephalon, contrasting with numerous iNOS-positive neuroglial cells. Contrarily, in dorsal telencephalon (neocortex and hippocampus), iNOS-positive neurons were plentiful, contrasting with the marked paucity of labelled neuroglial (astrocytes and microglial) cells. The dual distribution between iNOS-labelled neuroglial cells and iNOS-labelled neurons is a feature that has never been described before. Functionalities attached to such a divergent distribution are discussed.

## Introduction

Protozoan parasites belonging to the genus *Trypanosoma* are the causative agents of diseases affecting humans and animals. Human African trypanosomiasis (HAT) [1, 2] is transmitted by hematophagous tsetse flies that inject *Trypanosoma brucei* (*T*. *b*.) *gambiense* or *T*. *b*. *rhodesiense* resulting in two clinical entities. In both diseases, two stages occur successively. The early hemolymphatic stage 1 corresponds to parasite proliferation in the blood, lymphatic system and vital organs. The blood-brain barrier (BBB) crossing and the invasion of the central nervous system (CNS) by the parasites [1–3] constitutes the meningoencephalitic stage 2. The neurological signs of the disease are predominant during this stage, resulting in a wide panel of sensory, motor and psychiatric disturbances, along with sleep-wake cycle disruptions, which gave its name to the illness [2]. In animals infected with *T*. *b*. *brucei*, similar clinical disturbances have been described [3, 4].

Nitric oxide (NO) is a signaling molecule that exerts pleiotropic effects in different tissues. Synthesized by a family of four NO-synthases (NOS) including endothelial, neuronal, mitochondrial and inducible (iNOS) isoforms [5–7], NO has been involved in various physiological functions [5, 8–10] and pathophysiological processes including those related to parasite/host interactions [9, 11]. In our experimental model, NO production follows two opposite modalities: i) a decrease in the periphery (macrophages and blood) during both stages of the disease; ii) an increase in the brain during the meningoencephalitic stage 2 [9]. The early peripheral NO decrease reduces the trypanocidal pressure exerted by NO. This decrease is solely related to the iNOS isoform [9, 11]. It is primarily triggered by the secretome emitted by trypanosomes, which contains extremely active peptidases capable of inducing a rapid accumulation of asymmetric dimethylarginine, a potent iNOS inhibitor [9, 12]. Moreover, in the cascade of events triggered by the *T*. *b*. *brucei* secretome, the kinesin protein has been reported to shift the host immune cell arginine/NO metabolism from the NOS-dependent production of NO to that of arginase-dependent ornithine and derivatives (spermidine and glutathione) that are used by trypanosomes for their own growth through trypanothione synthesis [13, 14]. At this step, the peripheral innate immune system, deployed by the host to face the parasite (macrophages, B and T lymphocytes, NK cells, cytokines, NO and pyrogenic substances stimulating the immunity) [14], remains inefficient to counteract brain penetration by trypanosomes. In parallel, trypanosomes express a variant surface glycoprotein that helps them to escape immune reactions, disabling the production of antibodies characterizing the acquired form of immunity [15].

The cerebral compartment increase in trypanocidal NO production [9] is mainly due to the expression of dimethylarginine dimethylaminohydrolase-2 and to its ability to reduce the asymmetric dimethylarginine level [11]. The cerebral arginase activity remains unchanged throughout the infectious process, thus avoiding polyamine production that is known to promote trypanosome growth [11]. Therefore, brain mechanisms deployed to counteract trypanosome invasion are more efficient than those observed in the periphery. Nevertheless, the important rise in NO production resulting from the enhanced iNOS activity of astrocytes and microglial cells [9, 16] can be deleterious in reacting with superoxide anions to form peroxynitrites, thus triggering nitrosative and oxidative damages to proteins, lipids and deoxyribonucleic acid (DNA) [17]. Consequently, the neuroinflammatory response deployed by neuroglial cells (i.e., astrocytes and microglial cells) [18] to protect the infected brain by removing harmful stimuli may create conditions for neuronal vulnerability, e.g., myelin impairments, neurodegenerative processes and neuronal death [19, 20]. Despite the possibility that neuronal iNOS contributes to the deleterious oxidative stress, its actual implication is not fully understood. Neuronal iNOS may influence neuronal differentiation [18, 21], help tissue reparation through neurogenesis [22] or insure maintenance of basic functions such as sleep-wake alternation throughout the aging process [5].

To date, the precise identification of the cellular elements involved in brain inflammatory processes together with their biochemical substrate remain to be clearly determined. In order to further identify the anatomo-biochemical substrates expressed in brain inflammatory processes, Wistar rats were infected with *T*. *b*. *brucei*. In this respect, brain cells expressing iNOS (neurons and neuroglial cells) were identified in the infected animals and their brain distribution analyzed and discussed.

## Material and methods

### Experimental animals

All experiments were conducted in agreement with guidelines of the French Agriculture Ministry (Number: 03–505) and Agri-food of the forest (R-38UJF-F1-10). Experimental conditions to which the rats were submitted to Welfcare were also accepted by The Committee for Animal Experimentation of the Claude Bernard Lyon-1 University. Scientists holding an official position in the French research are required to acquire level 1 training in animal experiments and to update their training every 5 years. In the present investigation, R. Cespuglio, corresponding author, was responsible for compliance with the Ethical Standards.

The animals employed in this study were part of an anterior study deployed in the same experimental conditions [9, 11]. They were male Wistar rats (200–220 g) purchased from Janvier Laboratories (Le Genest Saint Isle, France) and housed in individual home cages at constant temperature (24 ± 0.5°C) and under a 12h light/dark cycle (light on at 5.00 a.m.; light off at 17.00 p.m.). The animals were provided with water and food *ad libitum*. After 8 days of adaptation to experimental conditions, they were divided in two groups, control (n = 5) and infected (n = 6) animals.

### Infection, parasitemia and brain tissue collection

**Fig 1:**
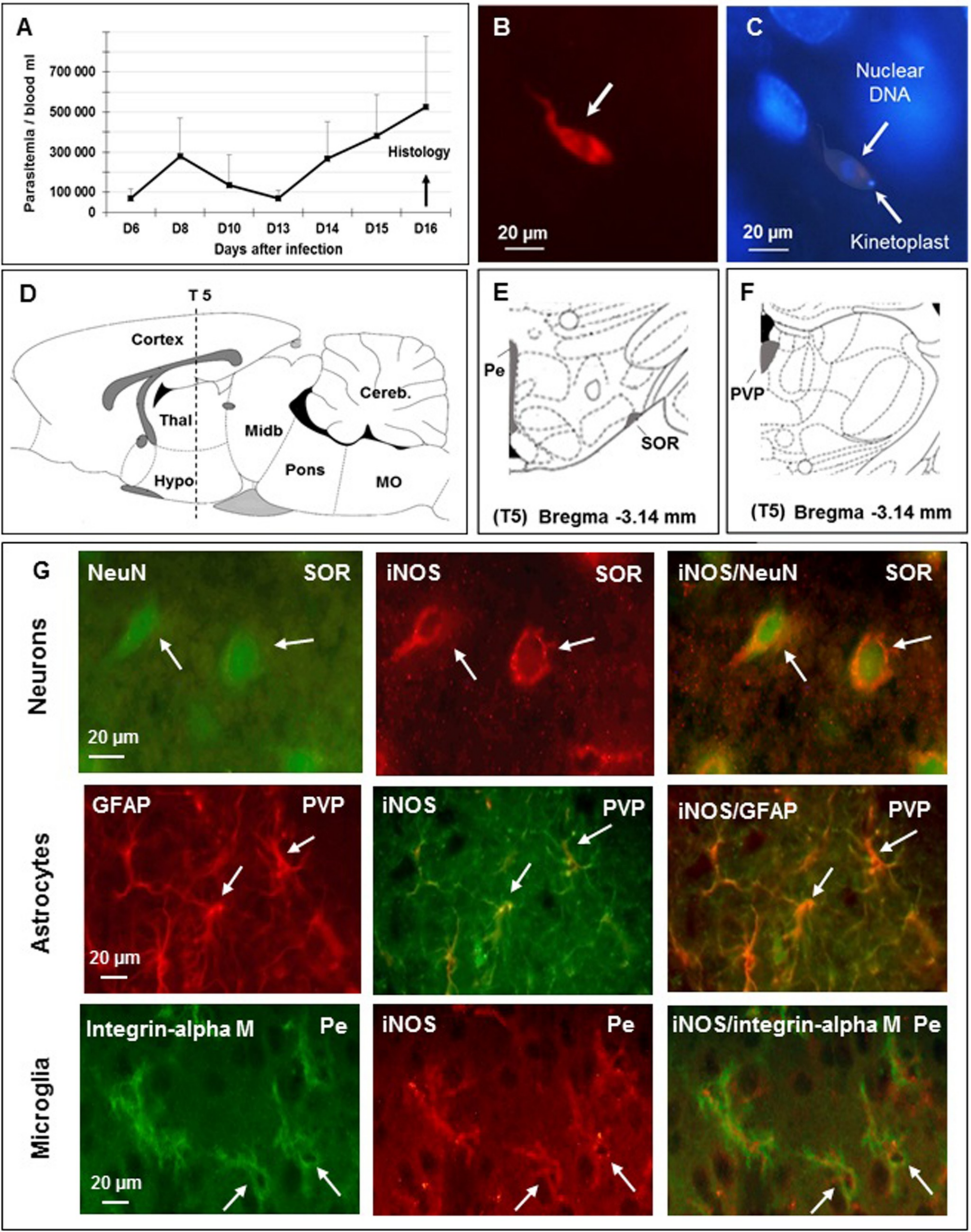
Identification of brain cells (neurons, astrocytes and microglial cells) expressing the inducible nitric oxide synthase (iNOS) in Wistar male rats 16 days after infection with *T*. *b*. *brucei*. **A–**Time course of parasitemia after infection (mean value ± SEM, n = 6, per day); trypanosomes appear in the blood 6 days (D6) later, corresponding to stage 1 human African trypanosomiasis (HAT); successive parasitemic waves take place; abscissae: days after infection; ordinates: parasitemia/mL. **B—**Immunofluorescent staining of *T*. *b*. *brucei* (in red, white arrow) in the brain parenchyma on D16 post-infection, corresponding to stage 2 of HAT (section S5, subfornical organ of the hypothalamus, n = 3), bregma -3.14 mm (according to reference [25]); **C**—Hypothalamic cell nuclei stained with DAPI (blue, subfornical organ of the hypothalamus); *T*. *b*. *brucei* are also stained (white arrows); **D**—Sagittal schema of the brain (lateral 0.4 mm / (according to reference [25]) showing the position of the 5^th^ coronal section (S5) chosen for iNOS immunostaining; **E and F**—Coronal schemas at bregma -3.14 mm (according to reference[25]) showing ventral (E) and dorsal (F) diencephalon. Grey matter, containing all brain cell types, was examined; **G—**Cellular labelling at section S5, bregma -3.14 using specific biological markers: neurons (NeuN), astrocytes (GFAP) and microglia (integrin alpha M) were identified (left column of part G); the 3 types of cells were also iNOS-positive (medial column of part G); double labelling appears in the right column (Neurons: NeuN and iNOS; Astrocytes: GFAP and iNOS; Microglia: Integrin alpha M and iNOS). **Abbreviations**: *T*. *b*. *brucei*, *Trypanosoma brucei brucei*; DAPI, 4’, 6’ Di amino-2-phenylindole; DNA, deoxyribonucleic acid; scale is given in μm, micrometer; NeuN, neuronal specific nuclear protein in vertebrates; GFAP, glial fibrillary acidic protein; Pe, periventricular hypothalamic n; PVP, paraventricular thalamic n, posterior part; SOR, supraoptic n, retrochiasmatic; An Olympus BX51 microscope was equipped with DP50 camera (objective 64x10).

Each rat was infected by intraperitoneal (i.p.) injection of 3,600 parasites in 200 μL of 0.9% saline solution (*T*. *b*. *brucei* AnTat 1.1E clone, provided by the Institute of Tropical Medicine, Antwerp, Belgium and stocked in calibrated cryotubes at -80°C). After 6 days, blood parasites were counted every two days from tail blood samples, using microscope with Malassez cell counting system (Fig 1). Sixteen days after infection, all animals were deeply anesthetized with chloral hydrate (400 mg/kg i.p.) and immediately submitted to an exsanguination by hearth infusion of Ringer lactate/heparin (0.05%) solution followed by a cold mixture (phosphate buffered saline, PBS, pH 7.4 containing paraformaldehyde at 4% and glutaraldehyde at 0.1%). Here, we underline that exsanguination was achieved under deep chloral hydrate anesthesia since an alternative anesthetic method (Ketamine/xylazine mixture), could not be employed. Indeed, we noticed that the ketamine/xylazine mixture, contrary to chloral hydrate, decreased drastically the activity of the iNOS enzyme as well as the quality of the immunohistochemical labelling. The negative influence exerted by the Ketamine/xylazine mixture on the iNOS activity was also confirmed by literature data [23, 24]. After exsanguination immediately followed by a decapitation, brains were removed and immersed overnight at +4°C in a fixative mixture of PBS pH 7.4 containing 4% paraformaldehyde and then immersed for 3 days at +4°C in 30% sucrose solution. After being frozen in 2-methylbutane (Sigma-Aldrich) at -40 to -60°C, frozen brain sections (25 μm thick) were obtained (head position according to [23]) and collected in PBS containing 0.3% Triton X-100 (PBS-T) with 2% hydrogen peroxide. After several rinses in PBS-T they were soaked (free-floating conditions) in PBS-T containing 0.01% sodium azide until use (Fig 1).

### Immunohistochemistry of iNOS

Following rinsing in PBS-T, brain sections were incubated with a mouse anti-iNOS monoclonal antibody (1/10000, Sigma-Aldrich) for 3 days at +4°C under constant agitation. After tissue washing, sections were incubated overnight (+4°C) in biotinylated horse anti-mouse (1/2000, Vector Laboratories, ABCYS, Paris, France). Following rinsing, sections were soaked for 2 h at room temperature in avidin-biotin complex (1/1000, Elite Kit, Vector Laboratories, Burlingame, CA, USA), and rinsed once more. The sections were stained in the presence of nickel, using 3, 3’-diaminobenzidine. Control sections were processed in the same way except that the primary antibody was omitted. All brain sections were mounted on slides, defatted and cover slipped. To add strength to the specificity of the anti-iNOS antibody employed in this study (as previously mentioned in [9]), peritoneal macrophages activated with LPS (0127-B8 and 0111-B4, 5 mg/mL, Sigma-Aldrich) were also employed as positive iNOS controls 8 and 24 hours after exposure to LPS. Moreover, since the iNOS western blot had already been achieved either in literature reports for the same rodent species [26] or by the anti-iNOS antibody supplier (Sigma-Aldrich, N9657, Immunoblotting, see catalogue: https://www.sigmaaldrich.com/catalog/product/sigma/n9657), we further analysed the activity of the iNOS enzyme and we measured NO, its end product. This approach could demonstrate that the iNOS protein expressed in brain tissue was responsible for the overproduction of NO since the neuronal form of the enzyme (nNOS) remained quite inactive [9].

### iNOS double labelling in neurons, astrocytes and microglial cells

We used mouse anti-iNOS monoclonal antibody (1/10000, Sigma-Aldrich), mouse anti-Neuronal Nuclei (NeuN) antibody conjugated with Alexa Fluor 488 (anti-NeuN, Alexa Fluor 488 conjugated, 1/500, Millipore, Molsheim, France), rabbit anti-Glial Fibrillary Acidic Protein (anti-GFAP) polyclonal antibody (anti-GFAP, 1/500, Dako Laboratories Carpentaria, California, USA) and mouse anti-integrin alpha M (OX42) FITC conjugated antibody (1/500, Santa Cruz Biotechnology, Dallas, Texas, USA). As secondary antibodies, we used a goat anti-mouse antibody conjugated with Alexa Fluor 488 or 568 (1/500, Invitrogen-Life Technologies, Cergy-Pontoise, France) and a goat anti-rabbit antibody conjugated with Alexa Fluor 568 (1/500, Invitrogen, Thermo Fisher Scientific, Waltham, Massachusetts, USA). Visualization was achieved using a Vecta-shield mounting medium with DAPI (nuclear stain, Vector Labs, Peterborough, UK) under fluorescence microscope (Fig 1).

### Trypanosome labelling

Anti-*T*. *b*. *brucei* AnTat 1.1E from an immunized mouse (1/400) and goat anti-mouse secondary antibody conjugated with Alexa Fluor 568 (1/500, Invitrogen) were used (Fig 1). Sera from healthy mice were used as controls.

### Cell count

In order to approach the density of labelled cells, visual counting (Olympus BX51microscope, objective 64x10) was achieved within 100-μm^2^ squares from a single brain section (to avoid double counting in successive brain sections). Such a procedure was performed in structures containing only iNOS-positive neuroglial cells (astrocytes and microglial cells, i.e., around the third ventricle: Pe, periventricular hypothalamic nuclei) or only iNOS-positive neurons (cortical areas: V2L, lateral part of the secondary visual cortices and CA1 Cornu Ammonis). Because of the variability of the labelling from one structure to another, the morphological changes taking place with individual infectious status, and to avoid false evaluations, counting was not performed in structures containing both iNOS positive neuroglial cells and neurons. Mean values ± SD are given.

## Results

### The iNOS-positive cell types

Trypanosomes appeared in the blood six days (D6) after the inoculation (corresponding to HAT stage 1). Two successive waves of parasitemia occurred at D8 and D16 post-infection. At D16 post-infection, extravascular trypanosomes were identified in brain tissues, primarily near ventricular cavities and basal brain areas (circumventricular organs, hypothalamic subfornical organ and median eminence; Fig 1), particularly during the waves of parasitemia. They confirm the development of a stage corresponding to HAT stage 2. Moreover, specificity of the cellular labelling was verified in areas containing the three cell types, i.e., periventricular hypothalamic nuclei (Pe), anterior part of paraventricular hypothalamic nuclei (PVA) and supraoptic retrochiasmatic nuclei (SOR) (Fig 1). Double labelling employing specific cell markers and iNOS antibodies, identified three types of cells, i.e., iNOS/NeuN for neurons in SOR, iNOS/GFAP for astrocytes in paraventricular thalamic-posterior part (PVP) and iNOS/integrin alpha M for microglial cells in Pe (Fig 1).

### Distribution of iNOS-immunostained cells in brainstem and cerebellum

**Fig 2:**
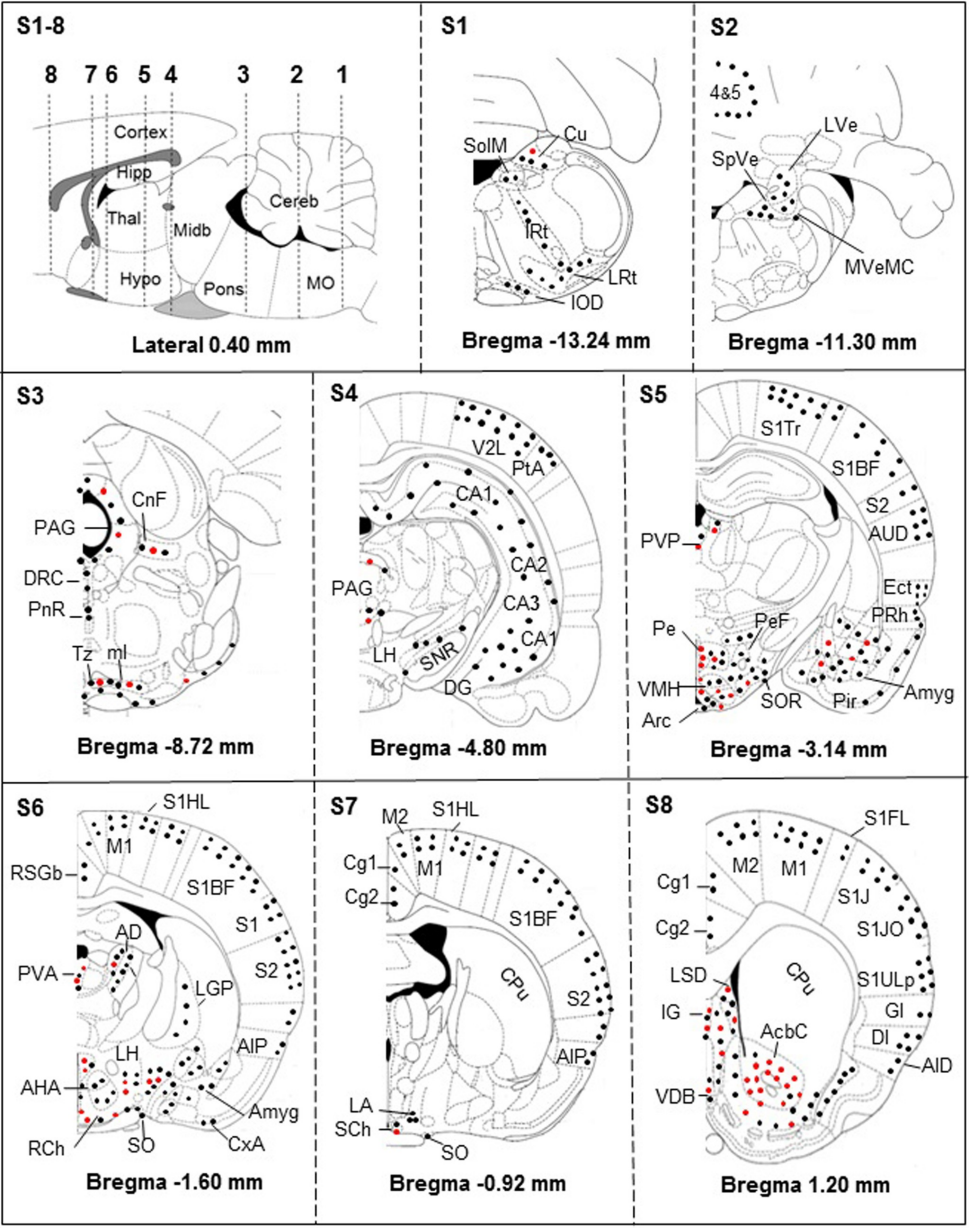
Brain distribution of iNOS-positive neurons (black dots), astrocytes and microglial cells (red dots). **S1-8** –Sagittal schema of the brain (lateral 0.4 mm; according to [25]) showing the 8 sections (S1-S8) performed in 4 rats. The sections extended from bregma +1.20 mm to bregma -13.24 mm and were analyzed for the distributions of the cells. The iNOS-positive astrocytes and microglial cells are mainly located in periventricular structures and basal parts of the brain. The cerebral cortex remains devoid of iNOS-positive astrocytes and microglial cells (S4, S5, S6, S7 and S8). **Abbreviations**: n, nucleus; 4&5, Cerebellar lobules; AcbC, accumbens n, core; AHA, anterior hypothalamic area, anterior part; AID, agranular insular cortex, dorsal; AIP, agranular insular cortex, posterior; Amyg, amygdala; Arc, arcuate n; AUD, secondary auditory cortex, dorsal; CA1, CA2, CA3, hippocampus fields (corni ammonis CA1, CA2 and CA3); Cortex, cerebral cortex; Cereb, cerebellum; Cg1, cingulate cortex, area 1; Cg2, cingulate cortex, area 2; CnF, cuneiform n; CPu, caudate putamen; Cu, cuneate n; CxA, cortex amygdala transition zone; DG, dentate gyrus; DI, dysgranular insular cortex; DRC, dorsal raphe n, caudal part; Ect, ectorhinal cortex; GI, granular insular cortex; Hipp, hippocampus; Hypo, hypothalamus; IG, granular insular cortex; IOD, inferior olive, dorsal n; IRt, intermediate reticular n; LA, lateroanterior hypothalamic n; LGP, lateral globus pallidus; LH, lateral hypothalamic area; LRt, lateral reticular n; LSD, lateral septal n, dorsal part; LVe, lateral vestibular n; M1, primary motor cortex; M2, secondary motor cortex; Midb, midbrain; ml, medial lemniscus; ml, Medial Lemniscus; MO, medulla oblongata; MVeMC, medial vestibular n, magnocellular part; PAG, periaqueductal gray; Pe, periventricular hypothalamic n; PeF, perifornical n; Pir, piriform cortex; PnR, pontine raphe n; PRh, perirhinal cortex; PtA, parietal association cortex; PVA, paraventricular hypothalamic n, anterior part; PVP, paraventricular thalamic n, posterior part; RCh, retrochiasmatic area; RSGb, retrospenial granular b cortex; S1, primary somatosensory cortex; S1BF, primary somatosensory cortex, barrel field; S1FL, primary somatosensory cortex, forelimb region; S1HL, Primary somatosensory cortex, hindlimb region; S1J, primary somatosensory cortex, jaw region; S1JO, primary somatosensory cortex, jaw region; S1Tr, primary somatosensory cortex, trunk region; S1ULp, primary somatosensory cortex, upper lip region; S2, secondary somatosensory cortex; SCh, suprachiasmatic n; SHi, septohippocampal n; SNR, substantia nigra; SO, supraoptic n; SolM, solitary n medial tract; SOR, supraoptic n, retrochiasmatic; SpVe, spinal vestibular n; Thal, thalamus; Tz, trapezoid body n; V2L, secondary visual cortex, lateral area; VDB, n of vertical limb diagonal band; VMH, Ventromedial thalamic. For a more detailed description of the structures, see [25].

Conventionally, the brainstem includes the mesencephalon (or midbrain), the metencephalon (or pons) and the myelencephalon (or medulla oblongata). Together, metencephalon and myelencephalon constitute the rhombencephalon (or hindbrain). In our approach, three caudorostral coronal sections (Fig 2) covered this part of the brain. The cerebellum (not included in the brainstem) lies posterior to the pons and medulla oblongata and is connected to the brainstem by three pairs of cerebellar peduncles (superior, middle and inferior). In our approach, this structure was covered by one coronal section (Fig 2). For simplification, only the most relevant histological illustrations are shown, as follows.

#### Section S1

**Fig 3:**
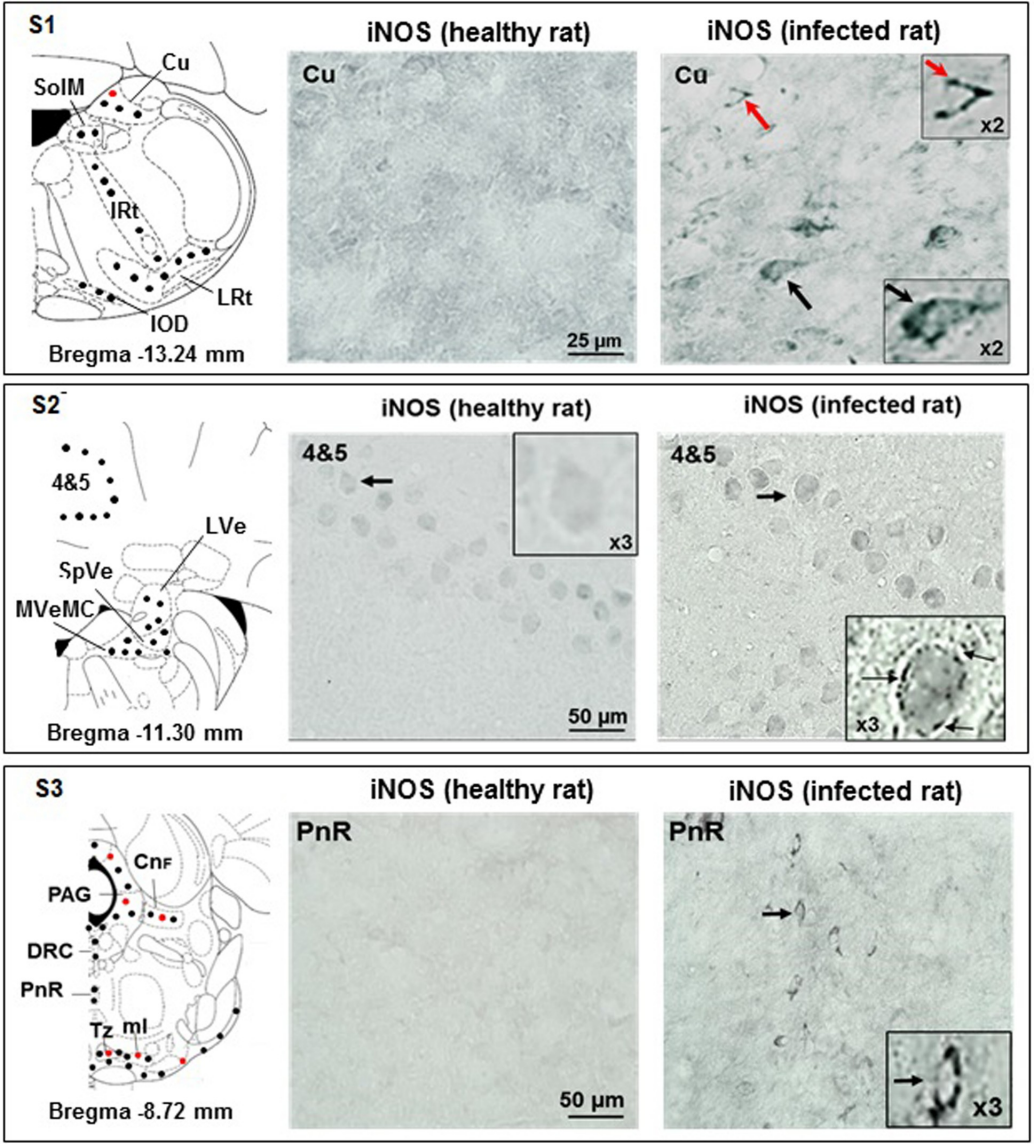
Immunohistochemical iNOS labelling observed in the brain slices obtained through sections S1, S2 and S3. **Section S1** (bregma -13.24 mm): absence of iNOS-positive cells in Cu area of control rats, contrary to infected rats (iNOS-positive astrocytes, red dots, and neurons, black dots); magnifications (X2) are shown; in infected rats, IOD, IRt, LRt, and SolM exhibit only iNOS-positive neurons (black dots; for histological data see File 1-Supporting Information). **Section S2** (bregma -11.30 mm): in control rats (also in X3 magnification), cerebellar lobule areas 4&5 do not reveal iNOS-positive cells; in infected rats, this area shows a layer of iNOS-positive Purkinje neurons (black arrows, also in X3 magnification); infected rats exhibit iNOS-positive neurons in LVe, MVeMC and SpVe (black dots in schema). **Section S3** (bregma -8.72 mm): in control rats, PnR is free of iNOS-positive cells, opposite to infected animals (iNOS-positive neurons, black dots, black arrows), who also exhibit DRC iNOS-positive neurons (black dots); infected animals, exhibit both iNOS-positive astrocytes (red dots) and neurons (black dots) in CnF, PAG, Tz, tz and ml. **Abbreviations and signs**: see Fig 2 and reference [25].

The iNOS was upregulated exclusively in infected rats; labelling was present in cuneate nuclei (Cu), both in neurons and neuroglial cells (Figs 2 and 3). Labelled neurons were exclusively present in the medial part of the solitary tract (SolM), the intermediate reticular nuclei (IRt), the lateral reticular nuclei (LRt) and the dorsal nuclei of the inferior olive (IOD) (Figs 2 and 3 and S1).

#### Section S2

The iNOS labelling appeared solely in neurons. Cerebellar neurons were labelled and, compared to healthy rats Purkinje cells, the labelling was weak but specific (pearled marking close to the membrane) (Figs 2 and 3). Labelled neurons were also present in the lateral vestibular nuclei (LVe), the spinal vestibular nuclei (SpVe) and the magnocellular part of the medial vestibular nuclei (MVeMC) (Figs 2 and 3).

#### Section S3

The iNOS labelling was observed in raphe system neurons, i.e., dorsal raphe caudal part (DRC) and pontine raphe nucleus (PnR, histological views shown) (Figs 2 and 3). Labelled iNOS-positive neurons, astrocytes and microglial cells were also observed in cuneiform nuclei (CnF), periaqueductal gray (PAG), trapezoid body (Tz) and medial lemniscus (ml) (Figs 2 and 3).

### Distribution of iNOS-immunostained cells in the diencephalon

The diencephalon includes the thalamus (Thal), the hypothalamus (Hypo), the epithalamus and the subthalamus. Four coronal sections (Fig 2) covered these structures, as follows.

#### Section S4

**Fig 4:**
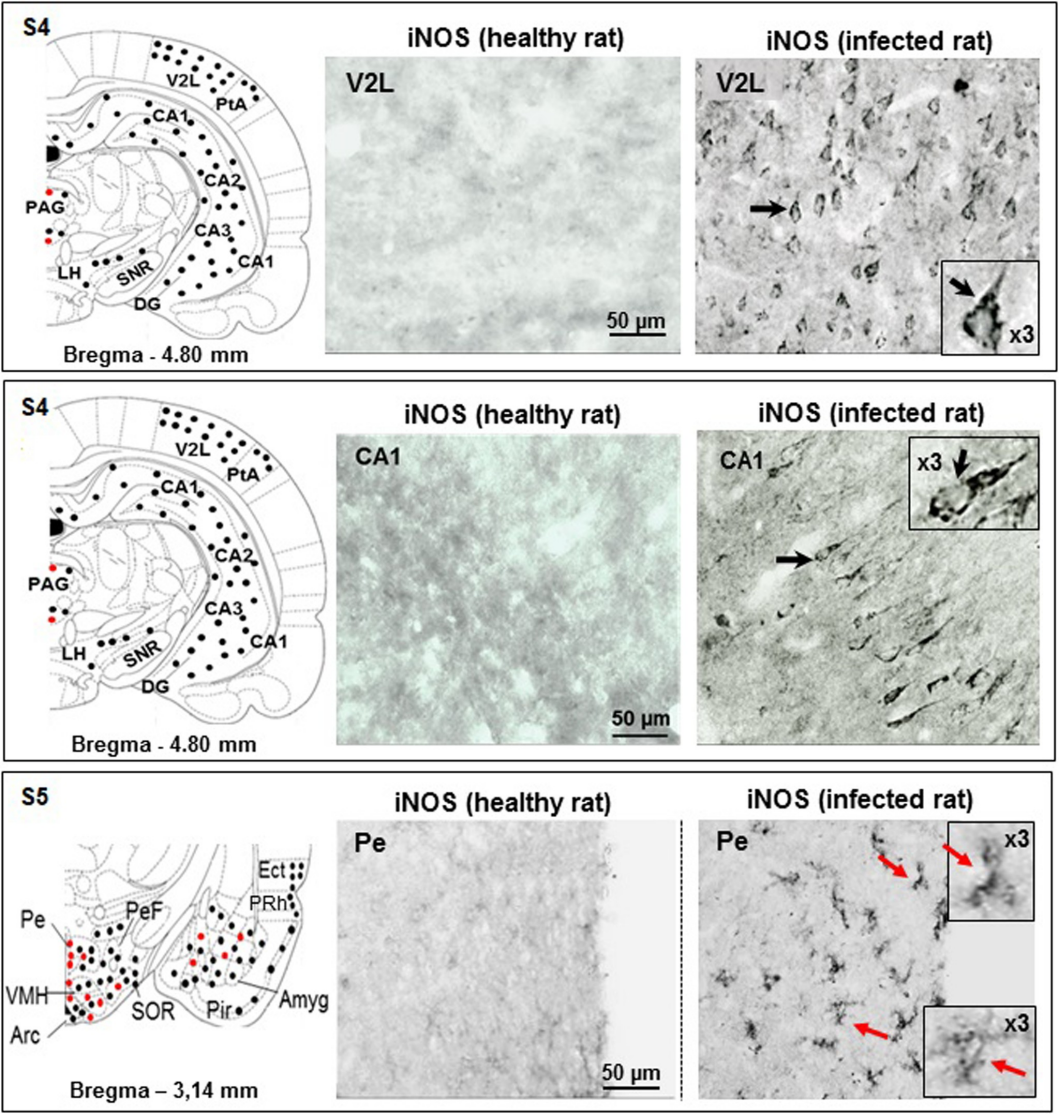
Immunohistochemical iNOS labelling observed in the brain slices obtained through sections S4 and 5. **Section S4** (bregma -4.80 mm): absence of iNOS-positive cells in V2L area of control rats, contrary to infected rats who exhibit iNOS-positive neurons (black arrow and dots); magnification (X3) is shown; in control rats, hippocampus CA1 area is free of iNOS-positive cell labelling; in infected rats, CA1 pyramidal neurons exhibit iNOS-positive labelling (black arrow, also in X3 magnification). Hippocampal (CA1, CA2, CA3 and DG) and cortical (PtA and V2L) areas exhibit only iNOS positive neurons (black dots). This is also the case for LH and SNR structures. iNOS positive neuroglial cells and neurons are both present in PAG (red and black dots). **Section S5** (bregma -3.14, ventral part): in control rats, Pe nucleus is free of iNOS-positive cellular elements, contrary to infected rats (Pe nucleus with labeled iNOS-positive astrocytes and microglial cells (red arrows, also in X3 magnifications); in the schema, Arc, PeF, VMH, SOR and Amyg exhibit mixed labelling (neurons, black dots, and neuroglial cells, red dots). Again, piriform (Pir) and ectorhinal (ECt) cortices exhibit only iNOS positive neurons (black dots). **Abbreviations and signs** see Fig 2 and reference [25].

In infected rats, the periaqueductal area (PAG) contained iNOS-labelled neurons and neuroglial cells (Figs 2 and 4). The lateral part of the hypothalamus (LH) exhibited only neuronal iNOS labelling (Figs 2 and 4). The histological views, V2L and CA1 in Fig 4, are discussed in telencephalic brain subdivision.

#### Section S5

Within the ventral part of S5, PVP contained iNOS-labelled neurons and neuroglial cells (Fig 2). Structures surrounding the third ventricular cavity, notably in Pe, only neuroglial cells were iNOS-positive (Fig 4). The ventromedial hypothalamic nuclei (VMH) housed a marked density of iNOS-labelled neuroglial cells (Figs 2 and 4). In the hypothalamic arcuate nuclei (Arc), neurons and neuroglial cells bore the iNOS labelling (Figs 2 and 4). When moving laterally, far from the third ventricle in the perifornical nuclei (PeF), only iNOS-positive neurons were observed (Figs 2 and 4). Laterally and ventrally, towards the SOR, iNOS-positive neuroglial cells became scarcer while labelled neurons were ubiquitous (Figs 2 and 4).

#### Section S6

**Fig 5:**
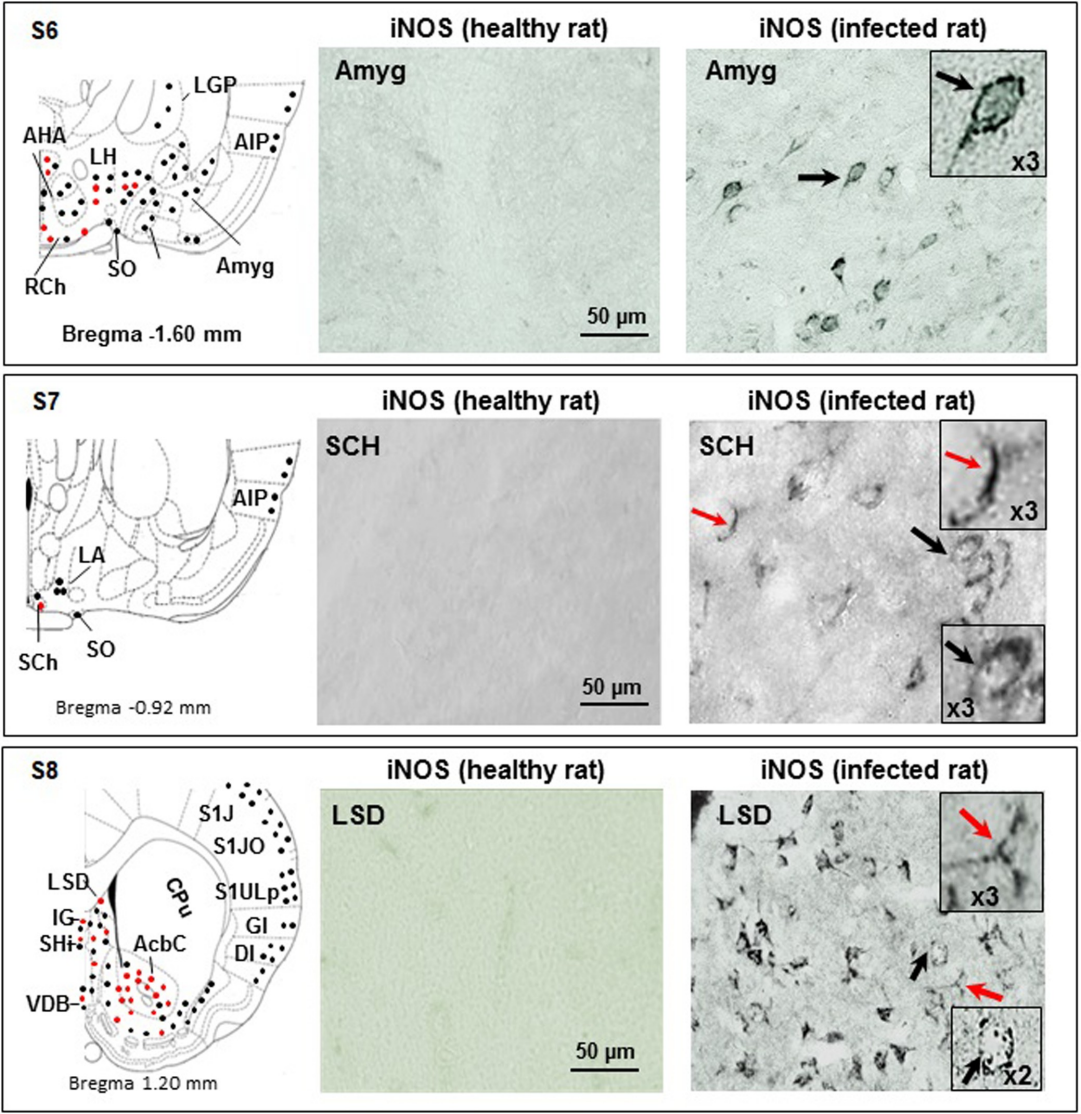
Immunohistochemical iNOS labelling observed in the coronal brain slices obtained through sections S6, S7 and S8. **Section S6** (bregma -1.60 mm): in control rats, Amyg is free of iNOS-positive cells; infected rats exhibit Amyg iNOS-positive neurons (black arrows and dots, also in X3 magnification); areas close to the 3^rd^ cavity (AHA, LH, RCh and SO) contain iNOS positive neurons and neuroglial cells (black and red dots); the LGP exhibits only iNOS positive neurons (black dots); the amygdalian cortex (CxA) and the agranular insular cortex (AIP) contain only iNOS positive neurons (black dots)**. Section S7** (bregma -0.92 mm): in control rats, the suprachiasmatic nuclei (SCh) do not reveal iNOS-positive cells; in infected rats, SCh exhibit iNOS-positive astrocytes (red arrow and dots) and neurons (black arrow and dots); magnifications (X3) are also shown; in LA and more superficial structure (AIP), only iNOS positive neurons are observed (black dots in schema). **Section S8** (bregma 1.20 mm): in control rats, the LSD does not reveal iNOS-positive cells, contrary to infected rats, who exhibit iNOS-positive astrocytes and microglial cells (red arrows and dots) and neurons (black arrows and dots). Magnifications (X3) are also shown; in the ventral part of the slice, iNOS positive neuroglial cells are abundant in AcbC and in a lesser extent in IG and VDB (red and black dots); again, superficial structures (AID, DI, GI, S1J, S1JO and S1ULp) exhibit only iNOS positive neurons (black dots). The CPu does not exhibit any iNOS positive cell. **Abbreviations and signs** see Fig 2 and reference [25].

Within the dorsal part of S6, the anterodorsal thalamic nuclei (AD), located below the lateral cavity, revealed iNOS-labelled neurons and neuroglial cells (Fig 2); in the anterior part of the paraventricular hypothalamic nuclei (PVA, just below the third cavity), iNOS-labelled neurons, astrocytes and microglial cells were present (Fig 2). Within the ventral part of section S6, iNOS-labelled neurons and neuroglial cells were observed in various hypothalamic structures, notably the lateral and anterior hypothalamic areas (LH, AHA; Figs 2 and 5). The histological view of Fig 5 is discussed in the amygdala sub-heading.

#### Section S7

Suprachiasmatic nuclei (SCh) contained iNOS-labelled neurons and neuroglial cells (Figs 2 and 5). Also, iNOS-labelled neurons were noticed in the supra optic nuclei (SO; Figs 2 and 5).

### Distribution of iNOS-immunostained cells in the telencephalon

This large brain area includes the neocortex, the hippocampal formation, the amygdala, septal nuclei, caudate nuclei, putamen structures and globi pallidi. The limbic system, which is a subdivision of the telencephalon, includes the hippocampal formation, the amygdala, the septal nuclei and specific cortical areas (cingulate, entorhinal, perirhinal and parahippocampal cortices).

#### Sections S4 to S8 for the neocortex

According to its large extension, the neocortex is present at different anteroposterior levels and is divided into six lobes: frontal, parietal, temporal, occipital, insular and limbic. The neocortex is also subdivided into 6 layers (I to VI, or 1 to 6, according to [25]). Layer 1, the molecular layer, consists mainly of apical dendritic tufts of pyramidal neurons and horizontally oriented axons, along with few scattered neurons. Layers 2 and 3 contain predominantly small and medium-size pyramidal neurons. Remarkably, throughout the coronal sections examined, the cortical iNOS labelling concerned only neurons of the superficial layers 1 to 3 (supragranular layers). In infected rats, the iNOS-labelled neurons were present in almost all cortical lobes, from the frontal to the limbic lobes (Figs 2, 4 and 5). The illustrations shown, i.e., the lateral area of the secondary visual cortex (V2L), indicate that pyramidal cells are quite exclusively responsible for the iNOS labelling (Fig 4).

#### Sections S4 and S5 for the hippocampus (archicortex)

This structure comprises the hippocampus proper (CA1, CA2 and CA3), the dentate gyrus, the subicular complex, and the entorhinal cortex. The hippocampus proper and the dentate gyrus exhibit only 3 layers, compared to the 6 layers of the neocortex. In the present study, the hippocampal formation (CA1) was examined mainly through brain coronal section S4 (Figs 2 and 4) and, in a lesser extent, through section S5 (Fig 2). In section S4, the iNOS labelling concerned particularly neurons present in CA1, CA2 and CA3 Cornu Ammonis (Fig 4). The histological illustration shown in Fig 4 indicates that pyramidal neurons of the middle layer of Cornu Ammonis (CA1) exhibited the iNOS labelling. To a lesser extent, iNOS-labelled neurons were also observed within coronal section S5, particularly in the limbic lobe, i.e., the ectorhinal (Ect) and the perirhinal (PRh) cortices (Figs 2 and S2). The iNOS labelling was also evidenced in neurons and neuroglial cells in the septohippocampal nuclei LSD (dorsal and ventral structures; Figs 2 and 5) and in the nuclei of the vertical limb diagonal band (VDB; Figs 2 and 5).

#### Sections S5 and S6 for the amygdala

The amygdala is located within the temporal lobe and is divided into three classes of nuclei: (i) the basolateral and basomedial nuclei; (ii) the cortical and lateral olfactory tract nuclei and (iii) the centromedial group, including the central and medial nuclei. A separate set of nuclei that represents the amygdalo-hippocampal transitional area (for detailed subdivisions see Fig 2 and reference [25]). In the basal and lateral parts of the amygdala, iNOS labelling concerned mainly neurons (Figs 2 and 5). In the most anterior part of the amygdala (Amyg, bregma -1.60 mm), only iNOS-labelled neurons were evidenced (Figs 2 and 5). These neurons exhibited a clear pearled marking close to the membrane (Fig 5). The amygdalian cortex of the transitional area (CxA) revealed also iNOS-labelled neurons (Figs 2 and 5).

### Other basal and basolateral structures

In the most rostral and basal part of the brain, the iNOS immunoreactivity observed in infected rats concerned particularly astrocytes and microglial cells and, to a lesser extent, neurons. This was the case for the accumbens nucleus that belongs to the basal ganglia (AcbC, core; Figs 2 and S3). Noticeably, two structures of the basal ganglia were devoid of iNOS-labelled neuroglial cells or neurons, i.e., the caudate putamen (CPu; Figs 2 and 5) and the substantia nigra (SNR; Figs 2 and 4). Structures of the dorsolateral pontine tegmentum did not contain iNOS-labelled neuroglial elements. Few iNOS-labelled neurons were present in the medial part of the pontine tegmentum (Figs 2 and 3).

### Density of the iNOS-positive cellular elements

As developed in the experimental procedures, a rapid count was achieved in structures where the iNOS labelling interested only neurons or neuroglial cells. In cortical areas and notably in the lateral part of the secondary visual cortex (V2L, Fig 4, infected rat), the iNOS was expressed only in neurons (6.33 ± 0.81 neurons/100 μm²; n = 6). In the hippocampal field and notably the Cornu Ammonis (CA1, Fig 4, infected rat), the iNOS was also expressed only in neurons (4.20 ± 0.83 neurons/100 μm²; n = 5). Around the third ventricle and particularly in the periventricular hypothalamic nucleus (Pe, Fig 4), iNOS was expressed only in neuroglial cells (5.16 ± 1.47 neuroglial cells/100 μm², n = 6). In order to circumvent approximate evaluations (variability in the neuroglial cell shape related to inflammatory status), counting was not achieved in structures containing both iNOS-positive neuroglial cells and neurons. In this respect, the histological views may reflect reliably the gradient of the changes taking place from the periventricular areas (100% of iNOS-positive neuroglial elements) to the cortical and hippocampal structures (100% of iNOS-positive neurons).

## Discussion

### Specificity of the iNOS labelling

The present manuscript reports data obtained from a group of rats that was part of previous investigations focused on cerebral and peripheral changes occurring in NO synthesis in a rat model of sleeping sickness [9]. The main finding of the study resides in the fact that an immunohistochemical expression of the iNOS protein occurred in Wistar male rat brains 16 days after infection with *T*. *b*. *brucei*, compared to healthy control animals. The immunohistochemical iNOS labelling was evidenced in neurons, astrocytes and microglial cells, but not in oligodendrocytes. Such an expression was not observed in healthy controls. The good specificity of the anti-iNOS antibody was also further controlled by achieving the positive iNOS control in macrophages activated by LPS with measurement of the iNOS enzyme activity [9]. Besides, literature data further underline that microglial cells, astrocytes and neurons possess and express the same iNOS protein [18]. These cells also express the transcription nuclear factor kappa B, NF-kappa B [27–29], a key regulator of inflammatory gene expression [17, 18]. Finally, at a post-infection time similar to the time at which the present immunohistochemistry was achieved, trypanosomes have been evidenced in the CNS [4, 9]. In this respect, we may therefore consider that our rats had reached a state of infection representative of neurological stage 2 of the human disease, HAT [1, 30].

### Brain distribution of iNOS-labelled neuroglial cells and neurons

According to Fig 2, distributions of neuroglial cells and neurons are not superimposable. Labelled neuroglial cells were mainly located in the basal forebrain, around the third ventricular cavity and, to a lesser extent, in the rostral part of the brainstem (periaqueductal gray, PAG) and its basal part. In such areas, iNOS-labelled neurons were scarce or absent. It is well known that trypanosomes are observed around the ventricular cavities (circumventricular organs, median eminence and subfornical organ of the hypothalamus) where the BBB and the blood-CSF (cerebrospinal fluid) barrier are the most permeable [31]. The prevalence of iNOS-positive neuroglial cells, able to produce marked amounts of NO, may rapidly exert NO-dependent trypanocidal activity [9]. Conversely, neuronal iNOS labelling was densest in telencephalic areas (cortical areas and amygdala), contrasting with the absence of iNOS-positive neuroglial elements. The distributions of both iNOS-positive neurons and iNOS-labelled neuroglial cells, may reveal a potential functional duality in response to trypanosome infection.

### Meaning of the iNOS expression

#### Microglial cells

In the CNS, glial cells/neurons ratio averages 10/1. Microglial cells represent the resident macrophages of the brain, i.e., its innate immune and inflammatory cells. They appear as small cells with a limited cytoplasm and branching [32]. Activated in response to pathogens and injuries, they proliferate rapidly, migrate to the infected site, phagocyte pathogens and remove damaged cells [33, 34]. Activated microglial cells may exhibit pro-inflammatory or anti-inflammatory phenotypes [35, 36]. They can produce and release pro-inflammatory cytokines such as interleukin-1 (IL-1), IL-6, tumor necrosis factor-alpha, and prostaglandin E2, but also anti-inflammatory cytokines (IL-4, IL-10) and transforming growth factor beta [29, 34, 37]. In various pathological situations, including trypanosomiasis, together with pro-inflammatory substances, microglial cells insure an important iNOS-dependent production of NO [37, 38 39]. Therefore, alike above reports, our data favor the capacity for a microglial production of inducible NO that exerts an innate and immediate trypanocidal activity.

#### Astrocytes

They are also part of neuroglial cells. Usually reported for being densest compared to microglial cells (3/1 ratio) [40], these star-shaped cells exhibit processes that may vary according to their brain location or functional state [41]. Astrocytes serve a wide range of adaptive functions in the mammalian CNS. Primarily, they produce lactate thus providing the main metabolic substrate for neurons [42]. Astrocytes play also a role in the uptake of neurotransmitters such as glutamate and gamma-aminobutyric acid, as well as in the regulation of extracellular potassium ion concentration [41]. Alike microglial cells, astrocytes express the iNOS and are capable to produce inducible NO. In pathological circumstances, NO produced by reactive astrocytes may modulate the blood flow towards a lesioned area, prevent the formation of epileptic foci or be protective as in encephalomyelitis [43]. Astrocytes can also be neuroprotective in releasing neuronal growth factors as well as soluble mediators, including IL-6, which have an impact on both innate and adaptive immune responses [41]. In the extracellular space, the astrocytic NO fraction joins the microglial NO fraction and also exerts a trypanocidal activity.

#### Microglial cells and astrocytes

They catalyze a high iNOS-dependent output of NO to challenge trypanosomes urgently. However, a “boomerang-like” effect is attached to this massive inducible NO release. Such a release may, indeed, speed up the vulnerability of other neuroglial cells such as oligodendrocytes (demyelination) and drive neurons to apoptosis [38, 43]. In our experimental conditions, the HAT experimental model exhibits an increased production of cerebral NO. Cell damage and neuronal signaling impairments result from NO cytotoxicity due to the combination of NO with the superoxide anion (O_2_^-^) to form various deleterious nitrogen oxides (NO_2_, N_2_O_3_) and peroxynitrites (ONOO^**-**^) [9]. These radicals are involved in the pathophysiology of various diseases including arthrosclerosis, arthritis, endotoxemia, respiratory distress syndrome and neurodegenerative diseases [9, 38, 44].

#### Neurons

In non-inflammatory conditions, neuronal NO is produced from L-arginine by the constitutive neuronal NO-synthase (nNOS). The activation of this enzyme is Ca^2+^/calmodulin-dependent. Calcium entry in neurons is regulated by N-methyl-D-aspartate (NMDA) receptors [45]. After synthesis, NO binds to the hem moiety of the cyclase and stimulates the synthesis of the second messenger cyclic guanosine monophosphate [46]. Constitutive NO is nowadays accepted as part of a new class of transmitters, the gaseous diffusible neuromodulators or messengers. In parallel, neurons are also capable of expressing iNOS. This calcium-independent enzyme is able to bind with calmodulin even at very low cellular concentrations of calcium. Consequently, iNOS activity does not respond to changes in cellular calcium content and is produced in neurons at lower levels than in neuroglial cells [47]. Such low NO levels are not suited for trypanocidal activity. The role of the iNOS expressed in neurons differs therefore from the function of neuronal NOS (neuromodulator/neurotransmitter) and that of neuroglial cells (trypanocidal in our experimental model). In experimental models of brain trauma free of LPS and cytokines susceptible of inducing non-specific responses [21], a *de novo* iNOS synthesis revealed by immunohistochemistry occurs in both neuronal and non-neuronal cells. At first, iNOS protein expression remains restricted to non-neuronal elements (4 hours). After 24 hours, iNOS expression appears in neurons [48]. The fact that the iNOS *de novo* synthesis occurs later in neurons versus glial elements further indicates that the neuronal NO fraction fulfils a different function. For example, in aged rats (20–24 months old), the sleep-wake architecture is maintained compared to adults (3–5 months old), despite specific changes including a progressive decrease in slow wave sleep and rapid eye movement (REM) sleep [5, 49]. In the aged animals, iNOS becomes expressed in neurons throughout the aging process. When iNOS expression is blocked with intraperitoneal delivery of specific inhibitors (2-amino-5, 6-dihydro-6-methyl-4*H*-1,3-thiazine, AMT; aminoguanidine, AG), REM sleep is entirely suppressed [49]. Therefore, in aging, the iNOS enzyme is progressively expressed in order to compensate the impairment of intercellular communication and/or key systems triggering sleep, i.e., cholinergic transmission and glutamate receptor activity [5, 49–51]. The recruitment of neuronal iNOS expression throughout aging may thus also exert a reparative function. Moreover, data from the present investigation show clearly that the brain distribution of iNOS-positive neurons is not superimposable to that of neuroglial cells. Such a distribution may result from the temporal dynamics of trypanosome invasion and subsequent damage of brain tissue. Early penetration of parasites takes place in structures located in the basal brain and particularly around the ventricular cavities where the BBB is weaker [31]. In such areas, the prevalent presence of iNOS-positive neuroglial elements, necessary to insure the trypanocidal NO release [9], also triggers the elaboration of deleterious nitrogen oxides (NO_2_, N_2_O_3_) and peroxynitrites (ONOO^**-**^) provoking neuronal damages and apoptosis [44]. As observed in aging, such deleterious effects may trigger the neuronal expression of the reparative iNOS enzyme.

## Conclusion

The present approach is first in achieving an original cartography of brain cells (neurons, astrocytes, microglia) expressing iNOS in rats infected with *T*. *b*. *brucei*. The iNOS-positive astrocytes and microglial cells exhibit a similar brain distribution in weak BBB areas (periaqueductal gray, surrounding the third cavity, basal telencephalon). The iNOS-positive neurons are distributed in different brain structures (dorsal part of the telencephalon), often located in areas that are poor or devoid of iNOS-positive astrocytes and microglial cells. Such distribution differences may serve different functionalities, i.e., a parasiticidal action for iNOS-positive microglial cells and astrocytes, versus protective and/or reparative roles for iNOS positive neurons. This aspect underlines the importance of animal models for diseases such as HAT, particularly in representing a general model for immune and reparation processes in the infected brain.

## Supporting information

S1 Fig Immunohistochemical iNOS labelling observed in the inferior olive (IOD, dorsal n.) through the coronal section S1 (bregma -13.24 mm). The schema on the left part of the figure shows the general distribution of iNOS-positive neuroglial cells (red dots) and neurons (black dots). In control rats (healthy rats) there is a total absence of iNOS-positive cells contrary to infected animals; in such animals, IOD, IRt, LRt, and SolM exhibit only iNOS-positive neurons (black dots). The illustration shown for IOD show the clear presence of labelled neurons (Black arrows). Such type of neuron is also observed in IRT, LRt and SolM. At the bottom of the figure, a neuron with an X3 magnification is shown. **Abbreviations and signs**: see Fig 2 and reference [25].
(TIFF)

S2 Fig Immunohistochemical iNOS labelling observed in the ectorhinal area of the cortex (Ect) through the coronal section S5 (bregma -3.14 mm). The brain coronal schema at bregma -3.14 mm (ventral part on the left of the figure) shows the general position of the labelled cells (red dots: neuroglial cells; black dots: neurons). In healthy control rats, the Ect do not reveal any iNOS-positive cellular elements. In infected rats, this area exhibits iNOS-positive neurons (black arrows). At the bottom of the figure, a neuron with an X3 magnification is shown. **Abbreviations and signs**: see Fig 2 and reference [25].
(TIFF)

S3 Fig Immunohistochemical iNOS labelling observed in the core of the nucleus accumbens (AcbC) through the coronal section S7 (bregma 1.20 mm). The brain coronal schema at bregma 1.20 (left part of the figure) shows the general position of the labelled cells (red dots: neuroglial cells; black dots: neurons). In healthy control rats, the AcbC do not reveal any iNOS-positive cellular elements. In infected rats, this area exhibit quite exclusively iNOS-positive neurons (red dots). At the bottom of the figure, a neuroglial cell with an X3 magnification is shown. **Abbreviations and signs**: see Fig 2 and reference [25].
(TIFF)
