# Supplementary figures and images for: Cerebral inducible nitric oxide synthase protein expression in microglia, astrocytes and neurons in Trypanosoma brucei brucei-infected rats
Source: PLoS One. 2019 Apr 17;14(4):e0215070. doi: 10.1371/journal.pone.0215070 (PMC6469759; doi:10.1371/journal.pone.0215070)

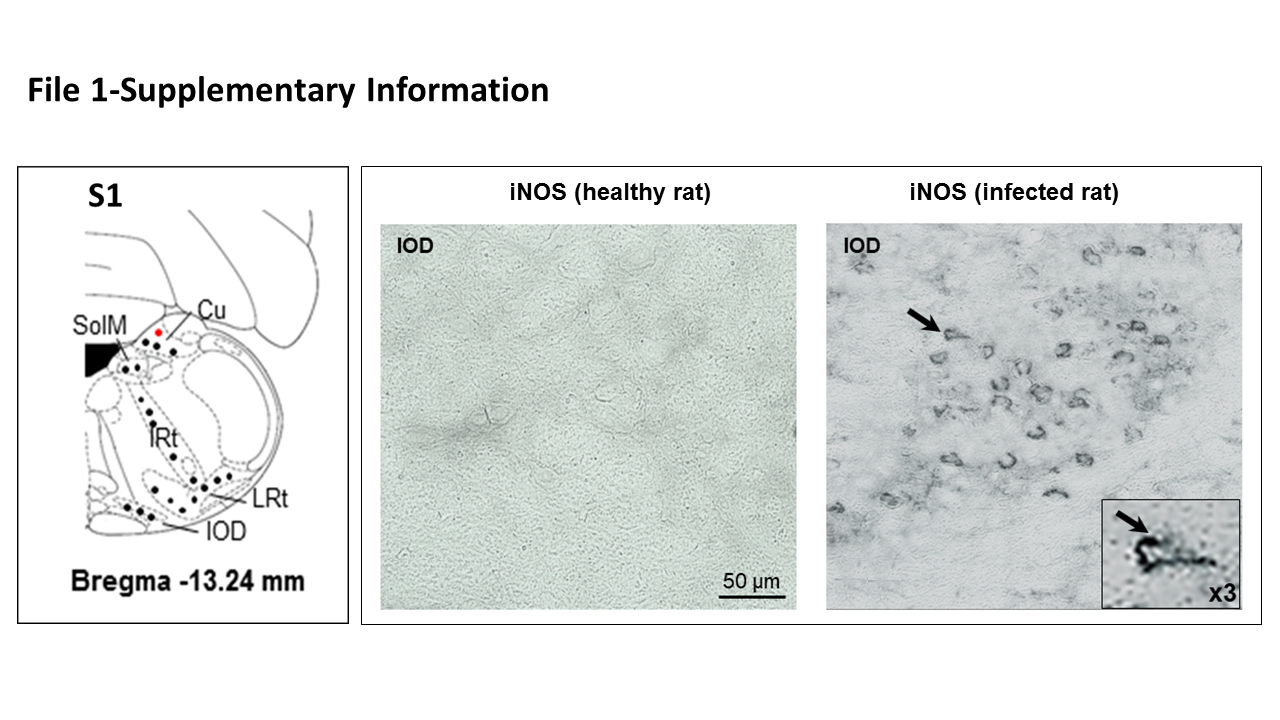

Supplement: S1 Fig — The schema on the left part of the figure shows the general distribution of iNOS-positive neuroglial cells (red dots) and neurons (black dots). In control rats (healthy rats) there is a total absence of iNOS-positive cells contrary to infected animals; in such animals, IOD, IRt, LRt, and SolM exhibit only iNOS-positive neurons (black dots). The illustration shown for IOD show the clear presence of labelled neurons (Black arrows). Such type of neuron is also observed in IRT, LRt and SolM. At the bottom of the figure, a neuron with an X3 magnification is shown. Abbreviations and signs: see Fig 2 and reference [25]. (TIFF) [file pone.0215070.s001.TIFF]

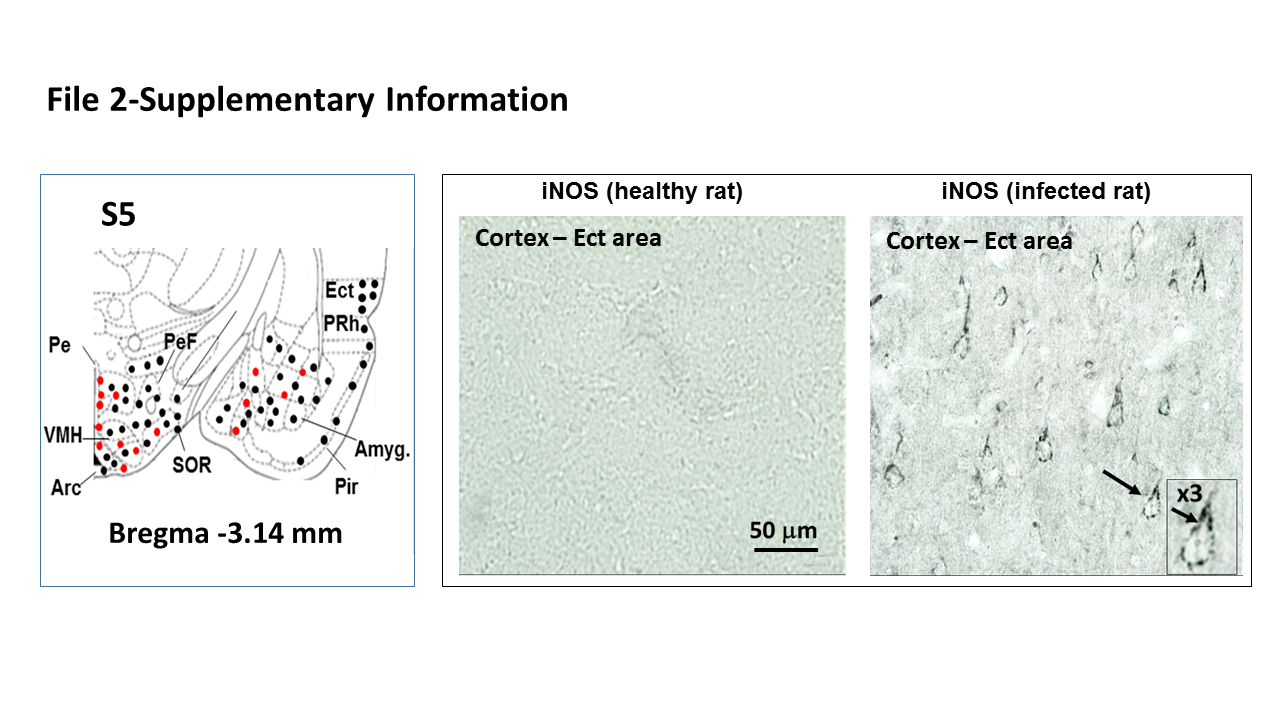

Supplement: S2 Fig — The brain coronal schema at bregma -3.14 mm (ventral part on the left of the figure) shows the general position of the labelled cells (red dots: neuroglial cells; black dots: neurons). In healthy control rats, the Ect do not reveal any iNOS-positive cellular elements. In infected rats, this area exhibits iNOS-positive neurons (black arrows). At the bottom of the figure, a neuron with an X3 magnification is shown. Abbreviations and signs: see Fig 2 and reference [25]. (TIFF) [file pone.0215070.s002.TIFF]

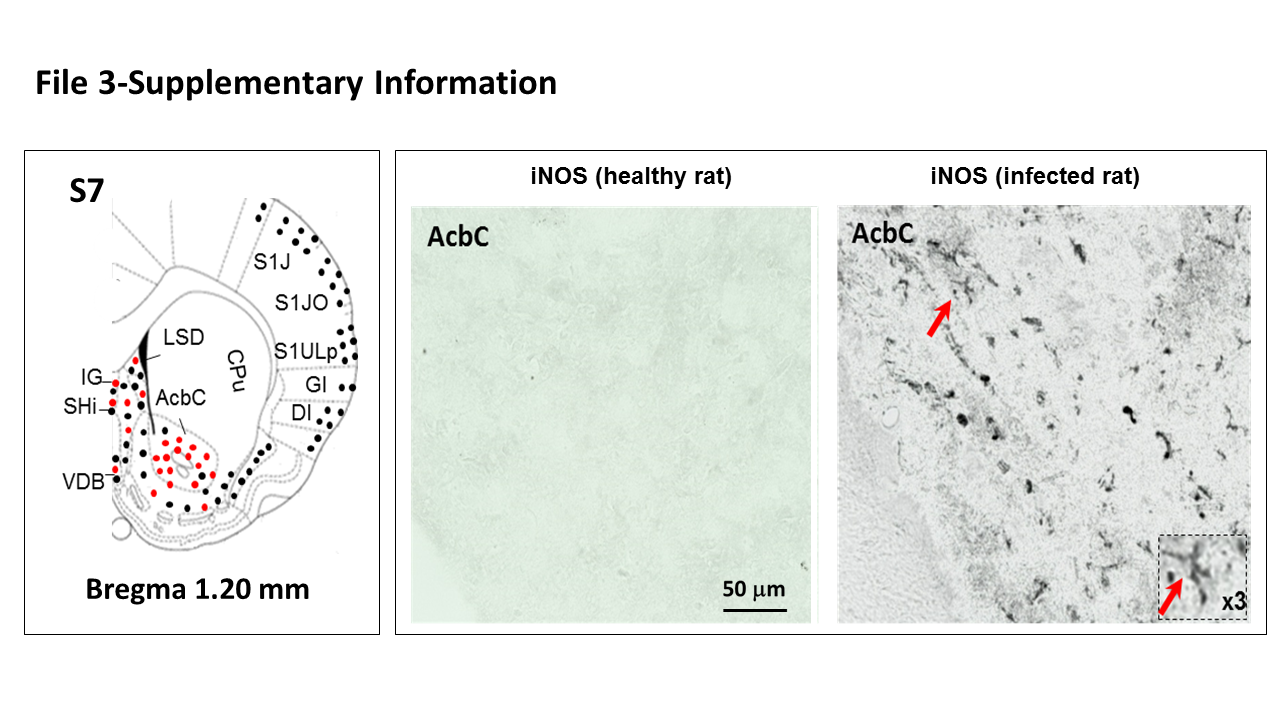

Supplement: S3 Fig — The brain coronal schema at bregma 1.20 (left part of the figure) shows the general position of the labelled cells (red dots: neuroglial cells; black dots: neurons). In healthy control rats, the AcbC do not reveal any iNOS-positive cellular elements. In infected rats, this area exhibit quite exclusively iNOS-positive neurons (red dots). At the bottom of the figure, a neuroglial cell with an X3 magnification is shown. Abbreviations and signs: see Fig 2 and reference [25]. (TIFF) [file pone.0215070.s003.TIFF]
